# Spontaneous haemothorax from an extramedullary haematopoietic mass

**DOI:** 10.1259/bjrcr.20150160

**Published:** 2015-11-12

**Authors:** Mahesh Kudari, Colin G Ferrett

**Affiliations:** Department of Radiology, John Radcliffe Hospital, Oxford, UK

## Abstract

We report the presentation of a 43-year-old female with an unusual acute complication from an inherited blood dyscrasia. After a provisional working diagnosis of pulmonary embolus, the patient was finally diagnosed with spontaneous haemorrhage from extramedullary haematopoietic foci within the thorax.

## Clinical presentation

A 43-year-old female presented to the emergency department with acute chest pain and shortness of breath 2 weeks after an uncomplicated emergency C-section. She complained of pleuritic type chest pain. An ECG did not reveal ischaemia or right heart strain but did demonstrate sinus tachycardia. Her history included β-thalassaemia major, splenectomy, cholecystectomy and mesenteric ischaemia. Supplementary oxygen was required at 2–4 l min^–1^ to maintain adequate oxygen saturation.

**Figure 1.**
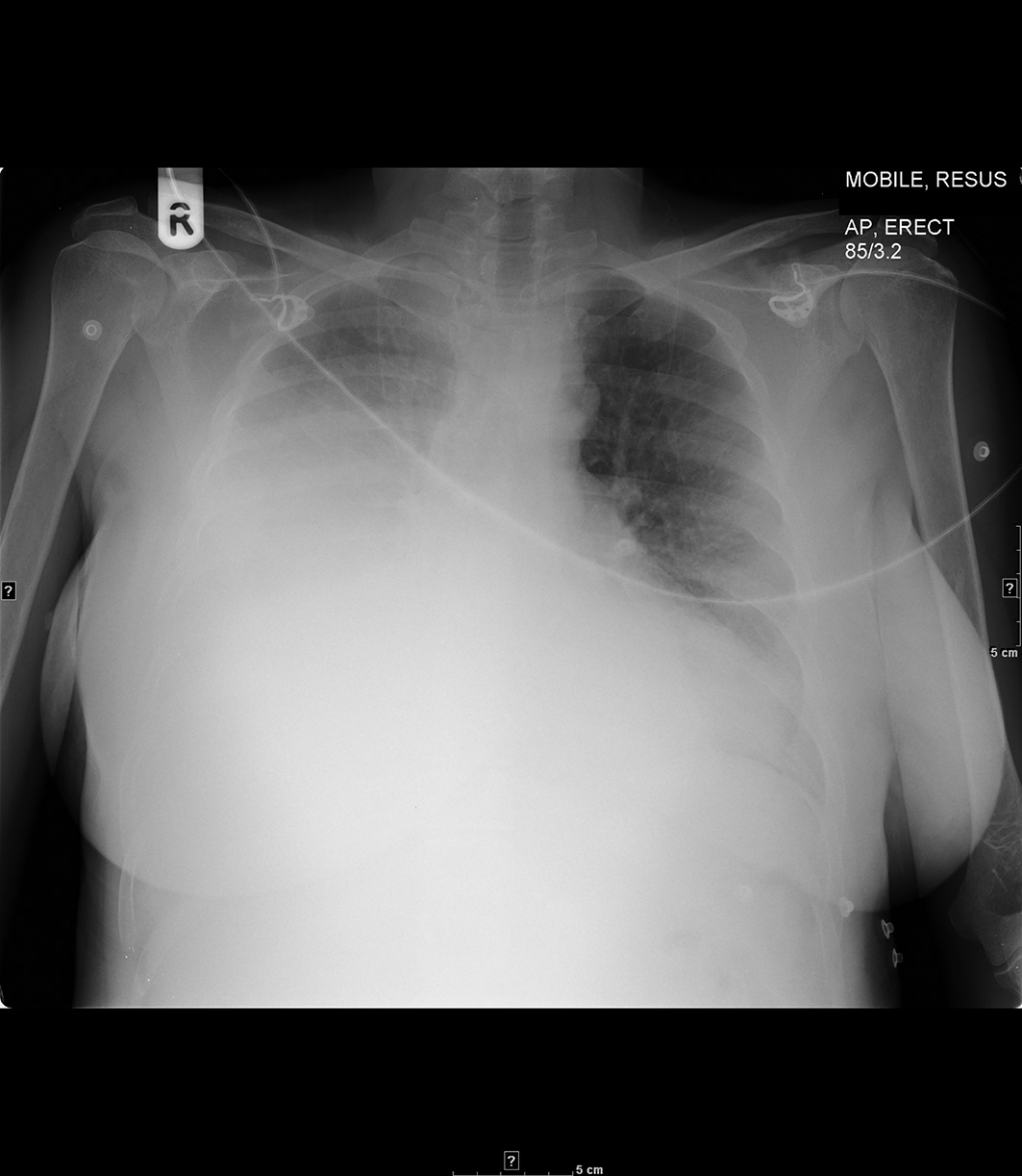
Chest X-ray at presentation shows a large right pleural effusion.

Given the nature of the pain, its acute onset and post-partum presentation, a pulmonary embolus was felt to be the most likely diagnosis and a chest X-ray was performed initially (Figure 1).

## Imaging findings

**Figure 2.**
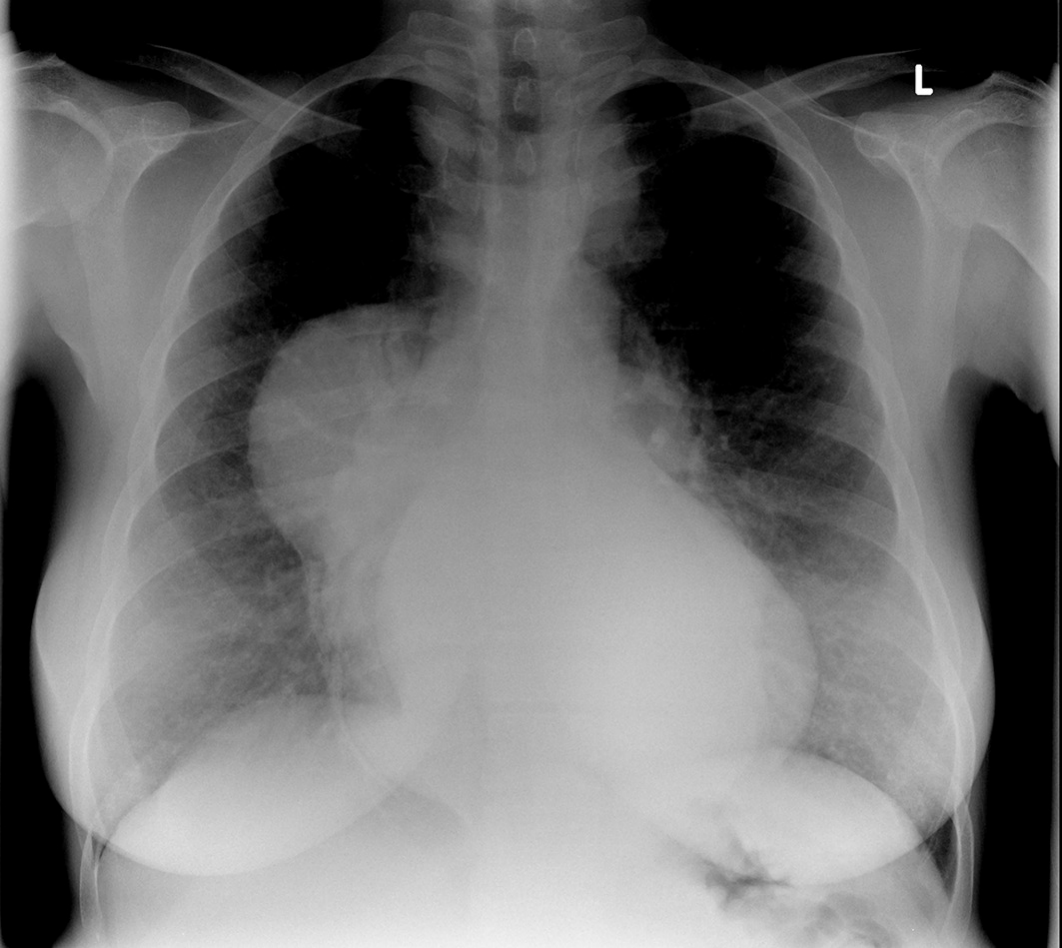
Review of a previous radiograph revealed paravertebral masses.

The radiograph revealed a large right pleural effusion, which was a new finding when compared with previous recent imaging. Further review of previous radiological examinations revealed pre-existing paravertebral and mediastinal masses, thought to represent haematopoietic tissue (Figure 2).

**Figure 3.**
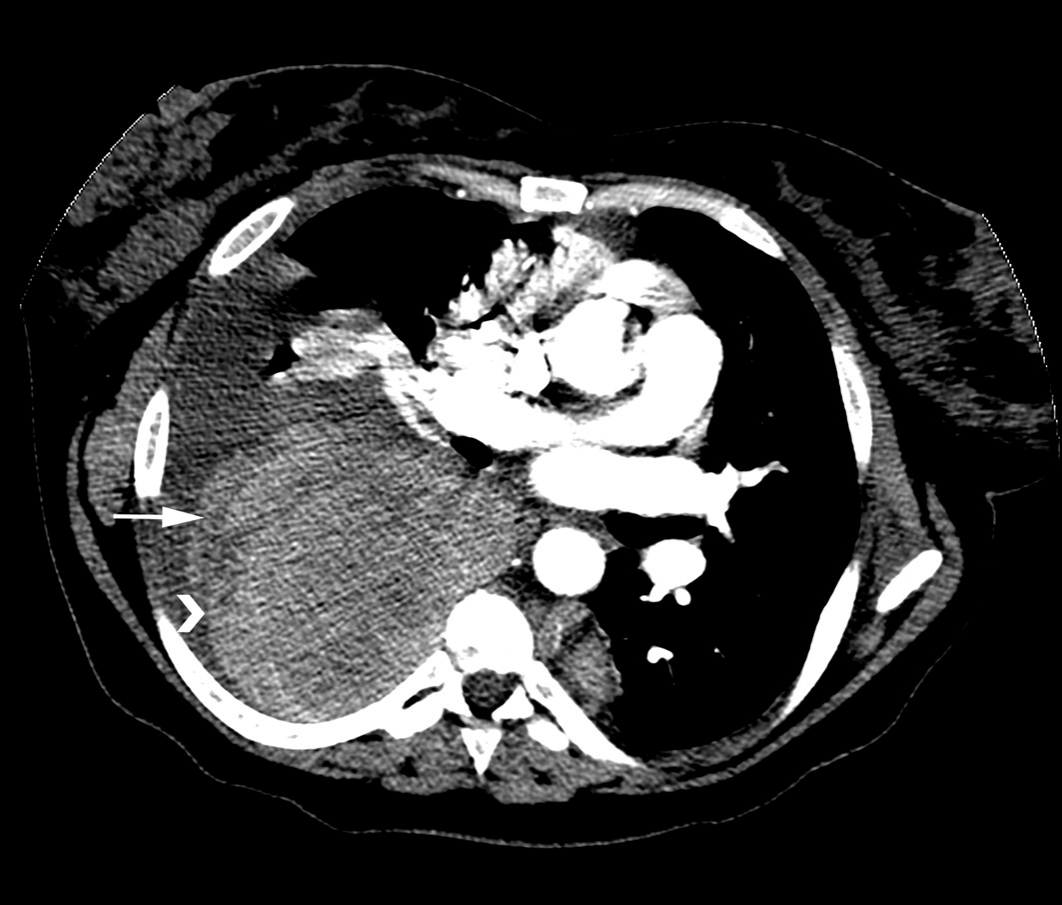
CT pulmonary angiogram at presentation showed layering haemothorax (arrow) adjacent to a large extramedullary haematopoietic mass (arrowhead) within the posterior mediastinum.

During the current hospitalization, a CT pulmonary angiogram (CTPA) was performed, which revealed layering of hyperdense material (arrow Figure 3) within the thorax, indicating haemorrhage from the haematopoietic masses (arrowhead delineates lateral border of haematopoietic mass; Figure 3).

## Treatment

After the CTPA, the patient became haemodynamically unstable with a low systolic pressure and surgical exploration was deemed necessary. Thoracotomy was performed and showed a mass with active haemorrhage from a parietal pleural tear and multiple similar lesions.

A large volume of haematoma was evacuated (1.5 l) from the pleural space. Histology from a sampled intrathoracic mass confirmed extramedullary haematopoietic tissue arising from a lymph node. The patient recovered after a blood transfusion and was discharged home after a few days with regular haematology follow-up.

## Discussion

Extramedullary haematopoiesis (EMH) is the production of blood cells outside the usual confines of bone marrow. It occurs as a compensatory response to chronic severe haemolytic anaemias (*e.g.* thalassaemia, sickle cell disease) and conditions where normal bone marrow function is diminished or lost (*e.g.* myelofibrosis and tumours).

Extramedullary haematopoietic foci have been noted in a wide range of sites—frequently the spleen, liver and lymph nodes, and occasionally within fat, adrenal gland, kidney, thymus, peripheral nerve, breast, cartilage, broad ligament, pleura, retroperitoneum, epidural space and epididymis.^1^^–^^3^

Such ectopic foci of haematopoietic activity are usually microscopic, although it is not uncommon for foci to form coalescent masses. Within the chest, EMH masses are typically paravertebral and within the posterior mediastinum, although they can also occur within the anterior and middle compartments.

The presence of EMH, even as large masses as in this case, is usually asymptomatic. When symptomatic, these highly vascular masses can present in many ways, including chest pain, fatigue, compressive symptoms on nearby structures and, not least, life-threatening haemodynamic compromise and respiratory failure.^4,5^

The patient reported here underwent a thoracotomy to evacuate the haematoma and stop the bleeding. Video-assisted thorascopic approaches have also been performed but in this case, better haemodynamic control could be achieved with open surgery. Longer term therapeutic approaches to reduce the volume of EMH have included transfusion–chelation therapy with drugs, such as hydroxyurea, and radiotherapy, particularly when there is compressive symptomatology. Where EMH has contributed to recurrent pleural effusion, strategies have included repeated thoracocentesis and pleurodesis.^6^

The imaging features of EMH have been reviewed previously.^3^ This case highlights the importance of recognizing EMH, as it can present as a life-threatening event, often years after the EMH masses form. In this particular case, review of the radiograph revealed a known presence of these masses. The chest radiograph (Figure 2) is exemplary in demonstrating the typical location of intrathoracic EMH and radiologists and other clinicians should be aware of the range of mediastinal masses, including EMH.

## Learning points

1. Chronic severe anaemia, from a wide range of causes, can lead to large foci of EMH. Unusually, these may rupture, leading to an acute life-threatening event.
2. When EMH is demonstrated on imaging, this should be highlighted for future awareness and as part of haematological monitoring and follow-up.
3. In an acute presentation, the chest radiograph can help characterize the location and the likely aetiology of mediastinal masses. Extramedullary haematopoietic masses are usually found in the posterior mediastinum, although occasionally in the anterior and middle mediastinal compartments. On cross-sectional imaging, extramedullary haematopoietic masses may contain fat.
